# Mixed‐type primary germ cell tumor of the mediastinum in a young adult male with a sudden life threatening condition: A case report

**DOI:** 10.1111/1759-7714.13231

**Published:** 2019-11-06

**Authors:** Tadashi Sakane, Katsuhiro Okuda, Takayuki Murase, Takuya Watanabe, Risa Oda, Tsutomu Tatematsu, Keisuke Yokota, Hiroshi Haneda, Hiroshi Inagaki, Ryoichi Nakanishi

**Affiliations:** ^1^ Department of Oncology, Immunology and Surgery Nagoya City University Graduate School of Medical Sciences Nagoya Japan; ^2^ Department of Pathology and Molecular Diagnostics Nagoya City University Graduate School of Medical Sciences Nagoya Japan

**Keywords:** Needl biopsy, choriocarcinoma, germ cell tumor, mediastinum

## Abstract

Primary germ cell tumors of the mediastinum are rare neoplasms. Above all, choriocarcinomas are highly aggressive with early haematogenous dissemination. Here, we report an extremely rare case of mixed‐type primary germ cell tumor of the mediastinum which occurred in a 26‐year‐old man with multiple metastases of the lung caused by choriocarcinoma components, with diffuse pulmonary hemorrhaging. The patient developed a sudden life threatening condition a few days after a needle biopsy.

**Key points:**

**Significant findings of the study**: This was an extremely rare case of mixed‐type germ cell tumor in a young adult male who developed a sudden life threatening condition due to choriocarcinoma components just a few days after a needle biopsy.

**What this study adds**: Serious conditions may occur in patients with germ cell tumor containing choriocarcinoma components. At present, there is no other way to treat such patients than to promptly recognize complications and perform urgent multimodal intervention.

## Introduction

Alhough extragonadal germ cell tumors (GCTs) are relatively infrequent, those occurring in the mediastinum are rare accounting for 3%–4% of all GCTs.^1^, ^2^, ^3^, ^4^, ^5^ Primary GCT of the mediastinum is sometimes aggressive and often has a poor prognosis, especially the nonseminomatous type.

## Case report

**Figure 1:**
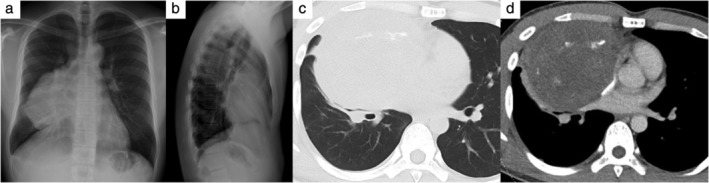
Chest radiography and computed tomography findings at the initial visit. Posteroanterior (**a**) and lateral (**b**) chest radiography findings showed an abnormal shadow in the right middle and lower lung field. Computed tomography findings revealed a well‐circumscribed tumor in the anterior mediastinum. (**c**) A pulmonary window image showed atelectasis in the right lung lobe. (**d**) Contrast‐enhanced computed tomography in a mediastinal window image revealed a heterogeneous tumor and some calcification.

A 26‐year‐old man presented to our hospital with a history of back pain lasting for six months. He had no past medical history or family history of note. A chest radiograph was taken which revealed an abnormal shadow at the right middle and lower lung field (Fig 1a,b). Chest computed tomography (CT) revealed an 11 × 10 × 9‐cm solid mass in the anterior mediastinum, representing a compressed superior vena cave and atelectasis in his right upper and middle lung lobe (Fig 1c,d). Laboratory analysis revealed an elevated white blood cell count of 12,500/μL (normal, >3,600/μL and ≤9,600/μL), lactate dehydrogenase (LDH) level of 278 U/L (normal, >119 U/L and ≤229 U/L), C‐reactive protein (CRP) level of 6.88 mg/dL (normal, ≤0.3 mg/dL), and a decreased hemoglobin level of 12.9 g/dL (normal, >13.2 g/dL and ≤17.2 g/dL). This analysis also revealed elevated serum tumor marker levels as follows: alpha‐fetoproteins (AFP) 450.7 ng/mL (normal, ≤10.0 ng/mL) and human chorionic gonadotropin (HCG)‐β 110 ng/mL (normal, ≤0.1 ng/mL). No masses were palpated in the testes.

**Figure 2:**
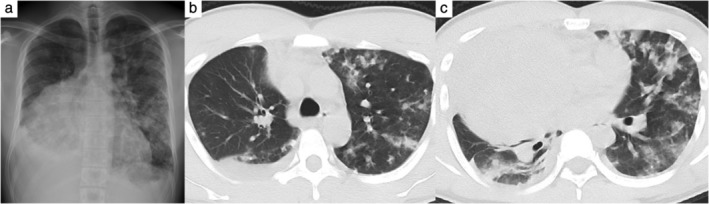
(**a**) Chest radiography in a patient with dyspnea and a high fever showed multiple mottled shadows in the bilateral lung. (**b** and **c**) Computed tomography showed multiple mottled shadows and pleural effusion in the bilateral lung.

Four days later, he was admitted to our hospital and underwent a percutaneous ultrasound‐guided needle biopsy of the tumor. He was discharged the day after the biopsy with no complications. However, two days after being discharged from hospital, he returned with dyspnea and a high fever of 40°C. A chest radiograph and CT revealed multiple mottled shadows in the bilateral lung and right dominant bilateral pleural effusion (Fig 2). Laboratory analysis revealed a markedly increased white blood cell count of 21,000/μL, LDH level of 426 U/L, and CRP level of 17.4 mg/dL. It also showed a decreased hemoglobin level to 11.0 g/dL. Antibiotics were commenced to improve the patient's general condition. However, his respiratory condition worsened day by day, and on the third day of hospitalization he was intubated because of hypoxemia. Bronchoscopy showed no tumorous lesions, but bleeding oozed out of the bilateral peripheral bronchus. Blood cultures and sputum cultures submitted at the time of readmission and on the third day of hospitalization were negative. The cytology of bronchoalveolar lavage was also negative. On the fourth day, the results of a histopathological examination of the tumor tissue obtained by the biopsy revealed potential malignancy, but a definitive diagnosis could not be obtained. Despite multidisciplinary treatment, he developed disseminated intravascular coagulation syndrome and acute respiratory distress syndrome. After holding an urgent cancer board meeting, we decided to start combination chemotherapy with bleomycin, cisplatin, and etoposide based on the clinical diagnosis of GCT. However, his general condition further worsened, and he passed away on the ninth day of hospitalization.

**Figure 3:**
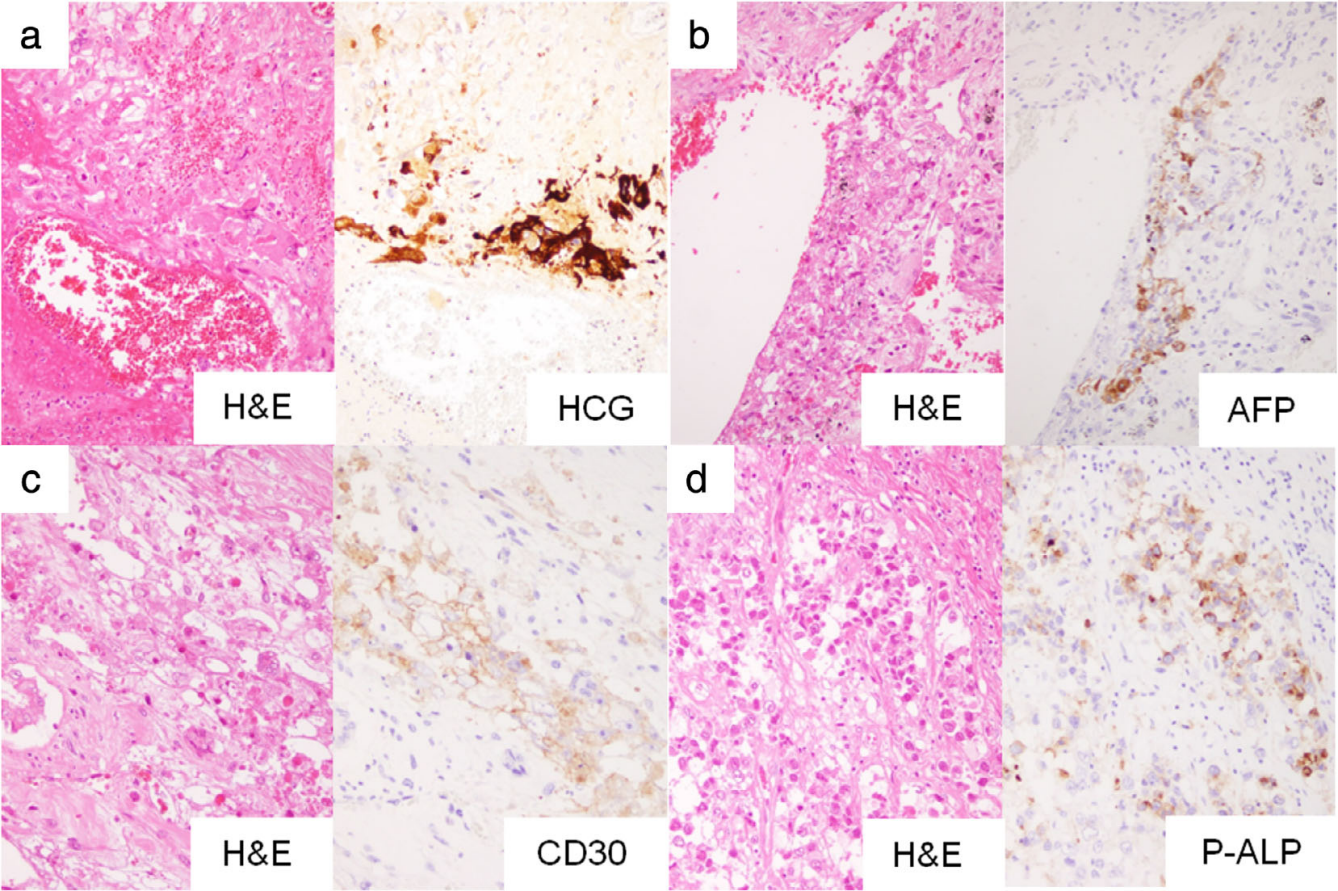
The mediastinal tumor consisted of four different types of histopathological architecture, confirming mixed‐type germ cell tumor. Representative hematoxylin and eosin (H&E)‐stained specimens and specific immunohistochemically stained specimens of each type. (**a**) Choriocarcinoma components. Large, bizarre, atypical cells were seen in hemorrhagic areas and were immunohistochemically positive for human chorionic gonadotropin (HCG). (**b**) Yolk sac tumor. Most tumor cells possessed eosinophilic cytoplasm, showed a relatively low nuclear‐cytoplasmic ratio, and were immunohistochemically positive for α fetoproteins (AFP). (**c**) Embryonal carcinoma components. Large, monotonous, atypical cells possessed eosinophilic cytoplasm and were immunohistochemically positive for CD30. (**d**) Seminoma components. Medium‐sized round tumor cells possessed poor cytoplasm and were immunohistochemically positive for placental alkaline phosphatase (P‐ALP).

**Figure 4:**
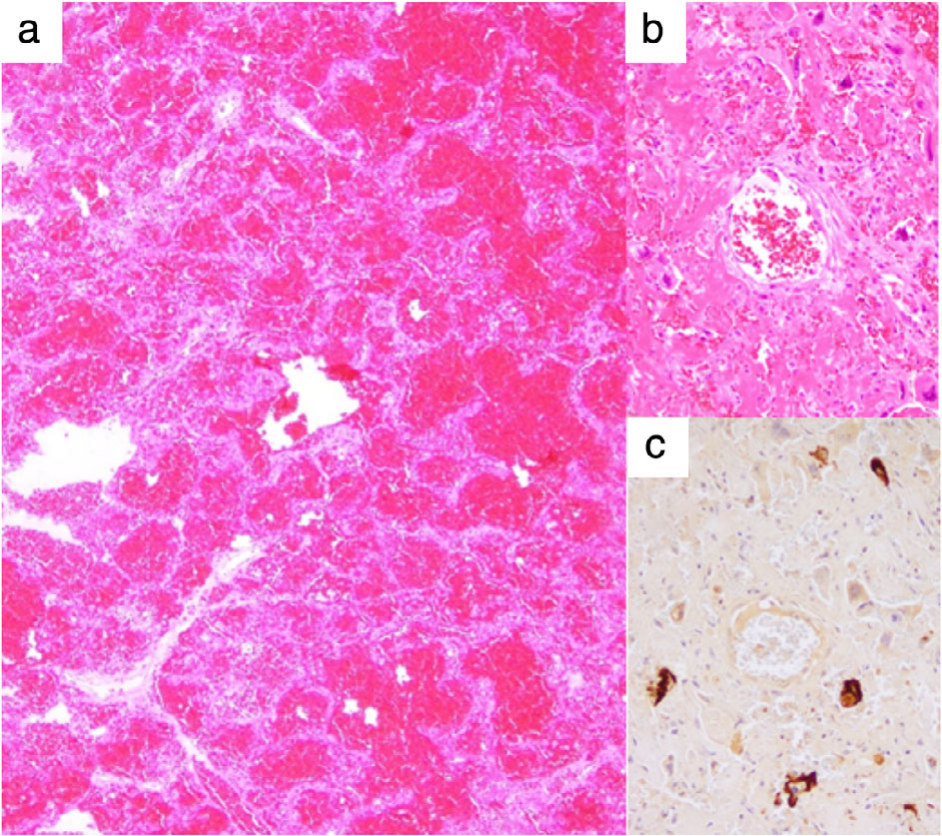
Both lungs showed diffuse and wide pulmonary hemorrhaging despite the small number of metastatic human chorionic gonadotropin (HCG)‐positive tumor cells. (**a**) A hematoxylin and eosin (H&E)‐stained specimen showing diffuse pulmonary hemorrhaging. (**b**) An H&E‐stained specimen showing a relatively small volume of tumor cells. (**c**) Tumor cells expressing HCG.

The autopsy revealed that the mediastinal tumor microscopically consisted of four different types of histopathological architecture, and each type was positive for specific immunohistochemical markers, suggesting mixed‐type primary GCT (Fig 3). No major vessels were involved by the tumor. Interestingly, wide and diffuse pulmonary hemorrhaging was microscopically observed despite the small volume of tumor cells that were positive for HCG suggesting choriocarcinoma (Fig 4).

## Discussion

In the present case, the patient suddenly experienced diffuse bilateral pulmonary hemorrhaging only a few days after the needle biopsy, resulting in death due to progressive and serious disease. In theory, it is therefore possible for tumor cells to enter the blood capillaries by a needle biopsy, leading to systemic metastasis. However, to our knowledge, this has not previously been reported. Although we may reasonably conclude that the needle biopsy was related to the patient's sudden deteriorization to a certain extent, whether or not the patient's worsening condition was caused by the needle biopsy is controversial.

We need to consider “choriocarcinoma syndrome” as an important condition similar to this case. Choriocarcinoma syndrome was first reported in 1984 by Logothetis and is characterized by hemorrhagic manifestations of metastases in advanced GCT containing large elements of choriocarcinoma.^6^ This syndrome is often brought about by the collapse of the tumor after chemotherapy and is characterized by a marked increase in the serum HCG‐β level.^7^, ^8^, ^9^, ^10^ In the present case, a histopathological examination revealed hemorrhaging mainly in the bilateral lung, despite a relatively small tumor which was not typical of choriocarcinoma syndrome. The present case may have characteristics that overlap in part with choriocarcinoma syndrome or may be a forme fruste of this syndrome; however, we feel that this does not entirely reflect a typical case of choriocarcinoma syndrome.

In our case, metastases into the lung were induced by choriocarcinoma. Choriocarcinoma commonly shows a mass composed of peripheral viable tumor cells with vascular invasion and central necrosis with hemorrhaging.^3^, ^11^ However, most of the lung lesions showed extensive and diffuse pulmonary hemorrhaging, despite the presence of only a small amount of metastatic tumor cells and necrotic tissue. Shintaku *et al*. reported an interesting case of primary choriocarcinoma of the lung manifesting in diffuse alveolar hemorrhaging and suggested that their case might be related to choriocarcinoma syndrome.^12^ Their patient showed progressive deterioration with diffuse alveolar hemorrhaging, resulting in death 13 days after the onset of initial symptoms, which was similar to our case.^12^ Their pathologic findings were diffuse and extensive fresh intra‐alveolar hemorrhaging without neoplasms.^12^ According to Shintaku *et al*. the destruction of the pulmonary vessels might be related to the tumor invasion and cause pulmonary hemorrhaging.^12^ In the present case, the tumor cells also might have been scattered beside the pulmonary vessels, causing pulmonary hemorrhaging. However, the mechanism underlying the diffuse and wide hemorrhaging was not clarified in the present case, even by microscopic examination.

We herein report a rare case of mixed‐type GCT of mediastinum with a life‐threatening clinical course. At present, there is no other way to treat such patients than to promptly recognize complications and perform urgent multimodal intervention. We must bear in mind that serious conditions may occur in patients with GCT containing choriocarcinoma components.

## Disclosure

The authors have no conflicts of interest to declare.
